# Single synchronous delivery of FK506-loaded polymeric microspheres with pancreatic islets for the successful treatment of streptozocin-induced diabetes in mice

**DOI:** 10.1080/10717544.2017.1377317

**Published:** 2017-09-15

**Authors:** Shiva Pathak, Shobha Regmi, Biki Gupta, Bijay K. Poudel, Tung Thanh Pham, Chul Soon Yong, Jong Oh Kim, Jae-Ryong Kim, Min Hui Park, Young Kyung Bae, Simmyung Yook, Cheol-Hee Ahn, Jee-Heon Jeong

**Affiliations:** a College of Pharmacy, Yeungnam University, Gyeongsan, Gyeongbuk, Republic of Korea;; b Department of Biochemistry and Molecular Biology and Smart-Aging Convergence Research Center, College of Medicine, Yeungnam University, Daegu, Republic of Korea;; c Department of Pathology, Yeungnam University College of Medicine, Daegu, Republic of Korea;; d College of Pharmacy, Keimyung University, Daegu, Republic of Korea;; e Engineering Research Institute, Department of Materials Science and Engineering, Seoul National University, Seoul, Republic of Korea

**Keywords:** Electrospray, FK506, microspheres, islet transplantation, immune suppression

## Abstract

Immune rejection after transplantation is common, which leads to prompt failure of the graft. Therefore, to prolong the survival time of the graft, immunosuppressive therapy is the norm. Here, we report a robust immune protection protocol using FK506-loaded microspheres (FK506_M_) in injectable hydrogel. Pancreatic islets were codelivered with the FK506_M_ into the subcutaneous space of streptozocin-induced diabetic mice. The islets codelivered with 10 mg/kg FK506_M_ maintained normal blood glucose levels during the study period (survival rate: 60%). However, transplantation of islets and FK506_M_ at different sites hardly controlled the blood glucose level (survival rate: 20%). Immunohistochemical analysis revealed an intact morphology of the islets transplanted with FK506_M_. In addition, minimal number of immune cells invaded inside the gel of the islet-FK506_M_ group. The single injection of FK506_M_ into the local microenvironment effectively inhibited immune rejection and prolonged the survival time of transplanted islets in a xenograft model.

## 1. Introduction

Type 1 diabetes is a metabolic disorder associated with autoimmune destruction of pancreatic β-cells in the islets of Langerhans. Thus, individuals with type 1 diabetes need life-long insulin therapy, which often leads to the occurrence of serious life-threatening complications (Bluestone et al., 2010). Pancreatic islet transplantation is an alternative method for the treatment of this disorder (Shapiro et al., 2000). However, the shortage of organs remains a major problem associated to islet transplantation (Shimoda & Matsumoto, 2017). To solve the problem of organ shortage, xenotransplantation has been proposed as an alternative method (Salama & Korbutt, 2017). In this regard, multiple studies have reported the successful transplantation of porcine islets in non-human primates (Cardona et al., 2006; van der Windt et al., 2009; Kim et al., 2016). Recent findings also have reported several attempts to transplant porcine islets into diabetic patients which appeared to show promising outcomes (Valdes-Gonzalez et al., 2005; Elliott et al., 2007). Since suppression of host immune cells is important for successful islet transplantation, development of xenogeneic islet transplantation protocol is a key for successful treatment of type 1 diabetes.

The portal vein is an ideal site for clinical islet transplantation. However, instant blood-mediated inflammatory reaction (IBMIR) at the injection site leads to the apoptosis of a large number of islets shortly after transplantation (Harlan et al., 2009, Naziruddin et al., 2014; Kourtzelis et al., 2015; Delaune et al., 2017). Injection *via* the portal vein is also associated with the embolization of the islets in the liver (Carlsson et al., 2001). In addition, being a rigorously invasive technique, portal vein transplantation exposes the patients to additional risks of hemorrhage, thrombosis, biliary puncture, transient rise in serum aminotransferase, and arterial-venous fistula (Shapiro et al., 1995). Thus, recent studies have explored the potential alternative sites for clinical islet transplantation including kidney, spleen, muscle, eye, peritoneum, omentum, thymus, bone marrow, and subcutaneous spaces (Rajab, 2010; Ali et al., 2016; Stokes et al., 2017a, 2017b). Islet transplantation into the subcutaneous space has a potential of clinical application because of the possibility to codeliver the cells and immune suppressive agents at the transplantation site.

Acute cellular immune response is another major cause for the loss of the graft shortly after transplantation (Hwang et al., 2017). In the past, authors have used combination therapies of various immunosuppressive drugs to increase the survival of transplanted organs. However, occurrence of life-threatening adverse effects and opportunistic infections attenuated the use of corticosteroids in solid organ transplantation (Anon, 1995). In 2000, Shapiro et al. reported corticosteroid free immune therapy called ‘Edmonton Protocol’ for successful islet transplantation in seven diabetic patients. Since then, therapeutic efficacy of clinically available nonsteroidal immunosuppressants such as FK506, rapamycin, mycophenolate mofetil, and cyclosporine A, have been widely explored (Shapiro et al., 2006). However, daily or repeated administration of the immunosuppressive drugs in clinical islet transplantation is often tedious and may result in patient noncompliance. Moreover, the systemic use of cocktailed immunosuppressive drugs aggravate adverse reactions on long-term administration (Shapiro et al., 2006). Therefore, use of prolonged release-type formulations for local immune suppression could be an ideal strategy to provide long-term immunosuppression after allo- or xenotransplantation. In this regard, numerous studies have reported the fabrication of drug-loaded prolonged-release type biodegradable polymeric microspheres (Amatya et al., 2013). Among the plethora of potential carriers, poly(lactic-co-glycolic acid) (PLGA) stands as one of the most common translational representatives for sustained drug delivery (Acharya & Sahoo, 2011; Sarisozen et al., 2017). Due to favorable degradation characteristics and possibilities for sustained-release drug delivery system (DDS), PLGA microspheres have attracted significant attention to encapsulate a range of hydrophobic as well as hydrophilic drug molecules. In addition, PLGA-based formulations reportedly achieved sustained release of drugs over a long period without significant fluctuations in plasma concentration profile (Miyamoto et al., 2004; Sevc et al., 2013). Recently, researchers have reported that the degradation of PLGA can be employed for prolonged release DDS by implantation without any invasive surgical procedures (Makadia & Siegel, 2011). Considering these facts, for the first time, we have incorporated FK506-loaded PLGA microspheres (FK506_M_) and pancreatic islets in an injectable hydrogel to provide effective immune suppression in mice model of type 1 diabetes.

In this study, we sought to design a system for synchronous delivery of pancreatic islets and FK506_M_ for transplantation into subcutaneous space of streptozocin-induced diabetic mice. Subcutaneous site is minimally invasive and avoids severe complications like IBMIR and thromboembolism associated with transplantation *via* the portal vein (Rajab, 2010). We codelivered pancreatic islets and FK506_M_ by a simple subcutaneous injection. The release of FK506 in the microenvironment of transplanted islets effectively inhibited the infiltration of immune cells without affecting the viability and functionality of the islets. Single administration of FK506_M_ significantly enhanced the graft survival compared with that of control. Thus, we suggest a robust therapeutic approach based on polymeric DDS for codelivery of pancreatic islets and FK506_M_ to improve the therapeutic outcome in pancreatic islet transplantation.

## 2. Research methodology

### 2.1. Preparation of FK506_M_

FK506_M_ were prepared using a single-nozzle electrospray machine as per the method described previously (Pathak et al., 2016a). FK506 was a generous gift from Hanmi Pharma Co., Ltd. (Seoul, Republic of Korea). Briefly, 15 mg of FK506 and 135 mg of PLGA (MW: 54 kDa) (Evonik Industries, AG, Darmstadt, Germany) were dissolved in 2 mL dichloromethane. The solution was then loaded into a syringe mounted on the electrospray machine to produce uniform-sized microspheres. To identify the drug encapsulation efficiency, a known amount of the microspheres, theoretically equivalent to 1 mg of FK506, was taken in a microtube, washed three times with distilled water, and centrifuged at 10,000 rpm for 10 min. Afterwards, 1 mL of acetonitrile was poured into the microtube to dissolve FK506_M_. Then, the solution was filtered, diluted appropriately, and quantified by using HPLC method, as described previously (Pathak et al., 2016a). Encapsulation efficiency (EE) was calculated using the following formula (Kondo et al., 2015; Seo et al., 2015; Zhao et al., 2015):

$$\text{EE }\left(\text{\%}\right)=\frac{\text{Total amount of FK}506}{\text{Actual amount of FK}506}\times 100\text{\%}$$

### 2.2. Solid state characterization of FK506_M_

Microspheres were evaluated based on morphological observations using scanning electron microscopy (SEM) (S-4100, Hitachi, Japan), X-ray diffraction (XRD) patterns using (X’Pert MPD diffractometer; PANalytical, Almelo, the Netherlands), thermal analysis using differential scanning calorimeter (DSC-Q200, TA instruments, New Castle, DE), and Fourier transform infra-red (FTIR) spectrometry using Nicolet Nexus 670 FT-IR spectrometer (Waltham, MA), as described previously (Pathak et al., 2016a).

### 2.3. Drug release study

The *in vitro* drug release study was performed in a media comprising of phosphate-buffered saline (PBS, pH 7.4; 1% v/v Tween 20) at 37 °C under sink condition in a water bath. Briefly, FK506_M_, equivalent to 1 mg of FK506, was placed inside a dialysis bag. The dialysis bag was kept in a tube containing 10 mL of the release medium. To perform the release study in the presence of the hydrogel, the microspheres were embedded in 500 µL of Matrigel (Corning, Bedford, MA), and placed inside the dialysis bag. Whole of the release medium was replaced every alternate day for analysis. To identify the kinetics of drug release, the release profiles with and without Matrigel were fitted to various equations described previously (Dash et al., 2010; Gupta et al., 2016).

### 2.4. Cytotoxicity studies

Cytotoxicity of blank microspheres was assessed using INS-1 cells by 3-(4,5-dimethyl-2-thiazoyl)-2,5-diphenyl tetrazolium bromide (MTT; Sigma-Aldrich, St. Louis, MO) assay, as described previously (Pathak et al., 2016b; Regmi et al., 2017).

### 2.5. Pharmacokinetics study

Pharmacokinetics study was performed in healthy male Sprague-Dawley (SD) rats. The rats were purchased from Samtako (Seoul, Republic of Korea), and housed under normal diet conditions. SD rats were used for the pharmacokinetics study because of the need of frequent blood sampling after injection. Briefly, FK506_M_ equivalent to 10 mg/kg FK506 was suspended in 500 μL of ice-cold Matrigel and injected into subcutaneous space over the flanks of the rats. Similarly, the rats in free drug group were injected with a suspension of 10 mg/kg FK506 in ice-cold Matrigel. Blood samples (100 μL) were withdrawn from the subclavian veins of the rats at specified time intervals. The blood samples were kept at −80 °C until further analysis. FK506 was extracted from the whole blood using methanol. Briefly, 100 μL of methanol was added to 100 μL of each blood sample, followed by three cycles (5 sec each cycle) of probe sonication at low amplitude. The lysate was centrifuged at 10,000*g* for 30 min. The supernatant was dried under neutral atmosphere of nitrogen to obtain a dry pellet. To prepare the sample for ELISA, each pellet was dissolved in 100 μL of phosphate-buffered saline (PBS; Hyclone, UT) containing 1% tween 20 using bath sonication. Measurement and calculation of whole blood FK506 concentrations were performed as per the manufacturer’s instructions using FK506 ELISA kit (Abnova, Taiwan).

### 2.6. Isolation of pancreatic islets

Healthy male SD rats weighing 250–300 g were used as islet donors. The experimental rats were housed under specific pathogen-free condition in animal care center of Yeungnam University (Gyeongsan, Republic of Korea). The rats were first anesthetized with a mixture of ketamine (90 mg/kg; Huons, Republic of Korea) and xylazine (10 mg/kg; Bayer, Republic of Korea) and then sacrificed by cervical dislocation. All the experimental protocols were strictly in accordance with the Institutional Animal Ethical Committee guidelines of Yeungnam University (IACUC 2016-014). Pancreatic islet isolation and purification were carried out as per the method described previously (Jeong et al., 2013). The islets were cultured in RPMI-1640 medium (Sigma-Aldrich) with 10% (v/v) fetal bovine serum (Hyclone), and 1% (v/v) penicillin/streptomycin (GenDEPOT454 Barker, TX). One day after isolation, the islets were washed, purified by handpicking, and cultured in fresh media. Islet transplantation was performed on day 3 of isolation.

### 2.7. Induction of diabetes and subcutaneous delivery of pancreatic islets

Diabetes was induced in C57BL/6 mice by single intraperitoneal injection of 200 mg/kg of streptozocin (STZ) (Sigma-Aldrich). To prepare the solution for injection, STZ was dissolved in ice-cold citric acid buffer (pH 4.5). Mice manifesting blood glucose level over 350 mg/dL for two consecutive days were selected as the diabetic recipients. For transplantation, four groups of mice were prepared as follows: (i) 2000 islet equivalent (IEQ) in Matrigel (islet-only), (ii) 2000 IEQ and FK506 powder (FK506_P_) (10 mg/kg) in Matrigel (islet–FK506_P_), (iii) 2000 IEQ and FK506_M_ (10 mg/kg) in opposite sites at the back using Matrigel (islet–FK506_M_#), and (iv) 2000 IEQ and FK506_M_ (10 mg/kg) at same site using Matrigel (islet–FK506_M_). To prepare the islets for injection, the Matrigel was thawed overnight at 4 °C. FK506_P_ or FK506_M_ was suspended with 500 µL ice-cold Matrigel containing islets and codelivered into the subcutaneous space over the flanks. For transplantation at different site, islets and FK506_M_ were separately suspended and transplanted on opposite sides over the flanks to ensure that the Matrigels containing islets and the microspheres do not come in direct physical contact. The group receiving islet using Matrigel without any immune suppressant was considered as the control. The injected hydrogel was allowed to solidify before the mice awoke. Transplantation was considered successful, if the non-fasting blood glucose level was maintained below 250 mg/dL for more than 2 days after transplantation. Non-fasting blood glucose levels were measured from the tail vein using portable glucometer. Non-fasting blood glucose level over 250 mg/dL for more than two consecutive days represented xenograft rejection. In addition, the body weight of the mice was measured at specific time points.

### 2.8. Intraperitoneal glucose tolerance test (IPGTT)

IPGTT was performed on day 14 and day 28 after transplantation to evaluate the responsiveness of the transplanted islets in islet–FK506_M_ transplanted mice. Transplanted mice were fasted for 8 h and administered with 2 g/kg of 20% glucose solution into the peritoneal cavity. Blood glucose levels were measured at 0, 5, 10, 15, 20, 30, 45, 90, and 120 min.

### 2.9. Hematological analysis

Various hematological parameters were checked using the serum samples withdrawn on the day of Matrigel retrieval. Blood urea nitrogen (BUN), creatinine (CRE), and electrolyte (sodium, potassium, and chloride) levels were measured using automated FUJI DRI-CHEM 4000i (Minato-ku, Tokyo, Japan). Briefly, blood samples were taken from the transplanted mice on the day of Matrigel retrieval, allowed to clot at room temperature for 30 min, and centrifuged to obtain supernatant containing serum. The serum was analyzed using the strips as per the manufacturer’s guidelines.

### 2.10. Quantification of insulin and cytokine levels in serum and Matrigel

Blood sample was collected on the day of Matrigel retrieval, kept at room temperature for 30 min to form clot, and centrifuged at 3000*g* for 20 min to separate the serum from the blood cells. The supernatant was then stored at −80 °C until further analysis. Insulin was quantified using rat/mouse insulin ELISA kit (Millipore Corp., Billerica, MA) and TNF-α was quantified using mouse TNF-α ELISA kit (eBioscience, Inc., San Diego, CA) as per the manufacturers’ instructions.

In addition, we measured the amount of insulin and TNF-α inside the Matrigel. A known weight of the Matrigel was homogenized in RIPA buffer (Thermo Scientific, Gangnam-gu, Seoul, Korea) to completely lyse the islets inside the Matrigel. Supernatant was collected after centrifugation at 6000*g* for 15 min at 4 °C and stored at −80 °C until further analysis.

### 2.11. Immunohistochemistry

We retrieved the Matrigel, fixed in formalin, and embedded in paraffin to prepare sections of 4 µm. The sections were then deparaffinized by heating in a dry oven for 1 h and washing vigorously in xylene. Rehydration was performed serially in 100%, 90%, 80%, and 70% alcohol. The antigens were retrieved by heating the slides in 10 mM citrate buffer of pH 6.0 using a microwave (5 min, 700 w). The citric acid was neutralized by immersing the slides in 3% hydrogen peroxide for 15 min. Slides were washed in PBS and incubated overnight at 4 °C with rabbit polyclonal anti-insulin (Abcam, Cambridge, MA), anti-CD3 (Abcam), and anti-CD68 (Abcam) in a humidified chamber. The slides were incubated at room temperature for 1 h. Then, the tissue sections were incubated with peroxidase-labeled secondary antibodies (Dako, Troy, MI). The slides were counterstained with hematoxylin and eosin, gradually dehydrated using 70%, 80%, 90%, and 100% alcohol, and fixed with a coverslip using mounting medium.

### 2.12. Statistical analysis

Statistical analysis was performed by using GraphPad Prism 5 (La Jolla, CA) and SigmaPlot version 12.0 softwares (San Jose, CA). Statistical values were calculated using unpaired *t*-test. Differences with *p* values of less than .05 were considered statistically significant.

## 3. Results

### 3.1. Preparation of FK506-loaded PLGA microspheres (FK506_M_)

FK506_M_ was prepared using electrospray machine (Figure S1(a)). Briefly, 135 mg PLGA and 15 mg FK506 were dissolved in reagent grade methylene chloride. The solution was filled into a standard syringe mounted over the electrospray machine. Upon application of electric field, the generation of electrostatic force inside the droplet led to the formation of microspheres (Figure S1(b)). The residual organic solvent was removed using a vacuum drier for 4 h. Drug encapsulation efficiency of the microspheres was 95.64 ± 1.37%.

### 3.2. Solid state characterization of FK506_M_

**Figure 1.**
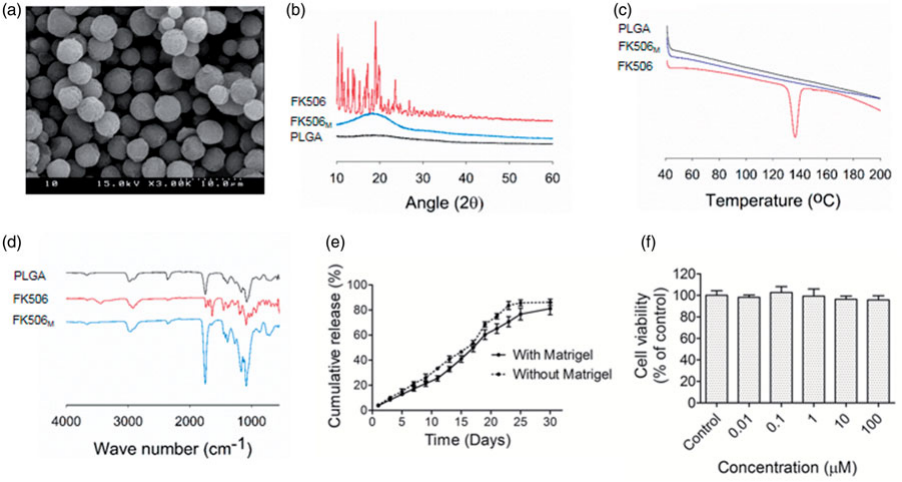
Preparation and characterization of FK506_M_. (a) Scanning electron microscopy, scale bar =10 μm. (b) X-ray powder diffraction (XRD). (c) Differential scanning calorimetry (DSC). (d) Fourier transform infrared spectrometry (FTIR). (e) In vitro release of FK506 from FK506_M_ in phosphate--buffered saline (pH 7.4) containing 0.5% Tween 20. The solid line indicates release profile of FK506 when the microspheres were incorporated with Matrigel and the dotted line indicates the release profile without Matrigel. The values represent means ± standard deviations (SD), *n* = 3. (f) *In vitro* cytotoxicity (MTT) assays of blank PLGA microspheres on INS-1 cells. The values represent means ± SD, *n* = 8.

Scanning electron microscopy (SEM) of FK506_M_ revealed a uniformly distributed spherical particles (Figure 1(a)). From the manual measurement of diameter using SEM software, the average size of the microspheres was approximately 5 μm (Figure S1(c)). The X-ray diffraction pattern showing the crystalline nature of FK506 was disappeared in FK506_M_, suggesting the incorporation of FK506 in amorphous state within the microspheres (Figure 1(b)). Sharp endothermic peak, akin to the one in the DSC of FK506 at around 140 °C was completely absent in case of FK506_M_, further indicating the amorphous state of the drug inside the microspheres (Figure 1(c)). The characteristic peaks of FK506 were observed in FTIR. No new peaks appeared in FK506_M_, indicating the absence of chemical bond formation during the incorporation of FK506 into the PLGA microspheres (Figure 1(d)).

### 3.3. *In vitro* release study of FK506_M_

The *in vitro* release study revealed a prolonged release profile of FK506 from FK506_M_, extending to more than 25 days (Figure 1(e)). Interestingly, the absence of burst release in the early days indicated an efficient incorporation of the hydrophobic drug inside the PLGA microspheres. Control of initial burst helps to avoid systemic toxicity, while sustained release pattern ensures long-term maintenance of constant therapeutic concentration. It is noteworthy that the presence of Matrigel did not significantly affect the release profile of the drug. However, the percentage of drug released in the early days was slightly lower in the Matrigel-incorporated FK506_M_. After fitting the release profiles of FK506 to different release kinetic model equations, coefficients of correlation (*r*^2^) were evaluated. A high degree of correlation with zero order equation (*r*^2^ with Matrigel and without Matrigel were 0.9691 and 0.9788, respectively) was observed. This indicated a concentration-independent release of FK506 from the microspheres.

### 3.4. Cytotoxicity of PLGA microspheres

The cytotoxicity of blank microspheres was evaluated by using rat insulinoma cells (INS-1). Data revealed no significant cytotoxicity on the cell line, suggesting the applicability of PLGA microspheres to deliver active agents to the cells (Figure 1(f)).

### 3.5. Pharmacokinetics of FK506 in SD rats

**Figure 2.**
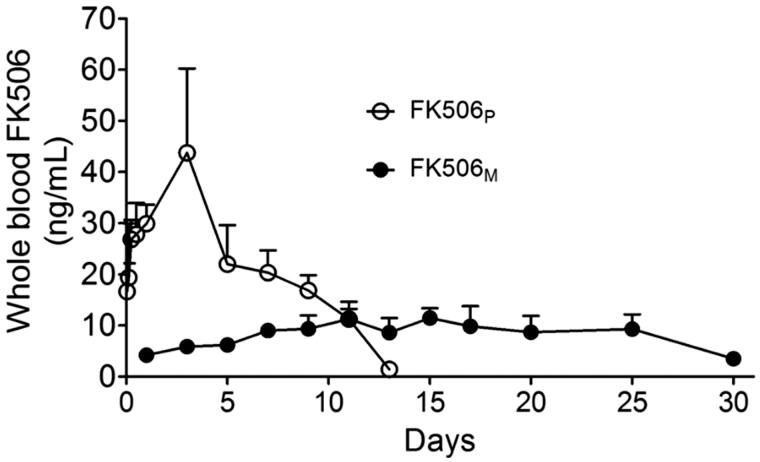
Pharmacokinetics study of FK506_M_ on male Sprague Dawley rats. The rats were subcutaneously injected with 10 mg/kg of FK506_P_ suspended in Matrigel (open circle, *n* = 4) and 10 mg/kg of FK506_M_ suspended in Matrigel (closed circle, *n* = 4). The values represent means ± standard deviations (SD).

Whole blood FK506 concentrations for FK506 and FK506_M_ groups are shown in Figure 2. FK506 was detected up to approximately two weeks in the FK506_P_ injected rats. The maximum drug concentration was 43.73 ± 16.49 ng/mL on day 3, which is sufficiently higher than the recommended therapeutic concentration of 10–20 ng/mL (Wingard et al., 1998; Przepiorka et al., 1999). In contrast, when the solution form of FK506 was injected into the subcutaneous space, FK506 immediately leaked into the circulation, reaching a maximum concentration (38.95 ± 14.70 ng/mL) at 8 h (Figure S2). FK506 was eliminated from the circulation in 24 h. Interestingly, FK506_M_ maintained a stable drug concentration approximately at the minimum recommended therapeutic levels. Having observed a long blood circulation of FK506, we speculated that the released drug could maintain a relatively higher concentration in the Matrigel and provided effective immune suppressive effects against infiltrating T-cells and macrophages without causing systemic toxicity.

### 3.6. Subcutaneous codelivery of islets and FK506_M_

**Figure 3.**
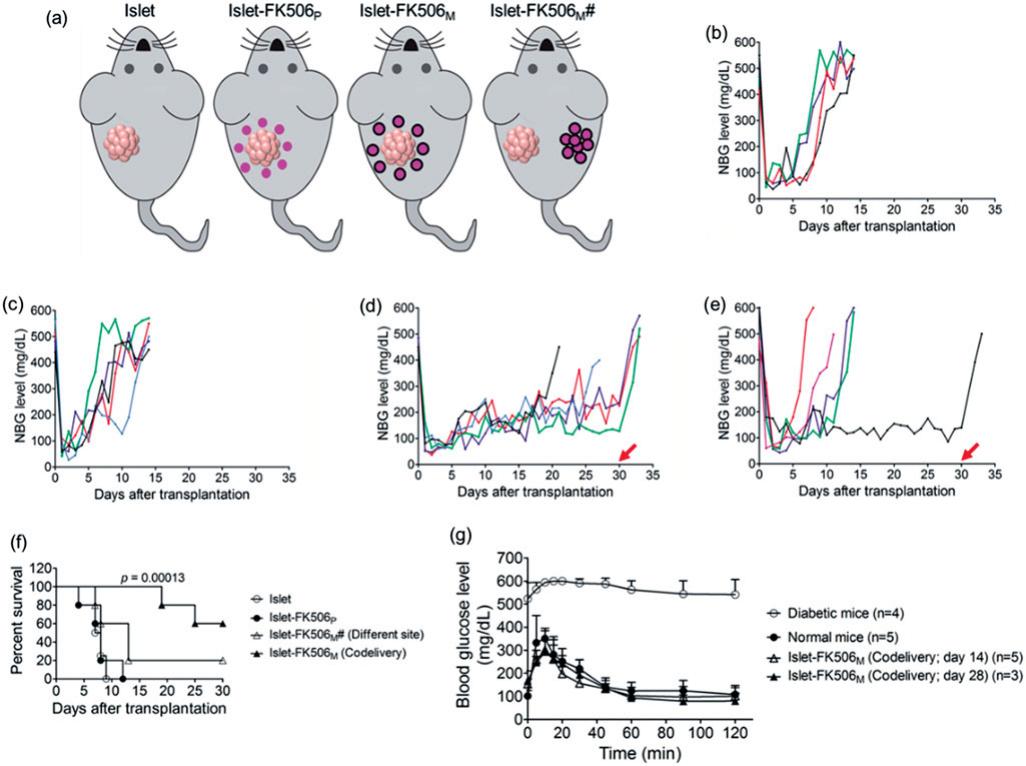
Transplantation of rat pancreatic islets in diabetic C57BL/6 mice. (a) Experimental groups. (b) Non-fasting blood glucose (NBG) level of the mice transplanted with islets (islet; *n* = 4). (c) NBG level of the mice transplanted with islets and FK506_P_ (10 mg/kg) at the same site (islet-FK506_P_; *n* = 5). (d) NBG level of the mice transplanted with islets and FK506_M_ (10 mg/kg) at the same site (islet-FK506_M_; *n* = 5). (e) NBG level of the mice transplanted with islets and FK506_M_ (10 mg/kg) on different sites (islet-FK506_M_#; *n* = 5). Arrows at day 30 indicate the day of retrieval of Matrigel. Each line indicates blood glucose profile of an individual mouse. (f) Kaplan–Meier curve for graft survival time. Graft survival time in islet-FK506_M_ increased significantly compared to that of the islet-only transplanted group (*p* = .00013). (g) Intraperitoneal glucose tolerance test (IPGTT) in diabetic mice (open circle; *n* = 4), normal mice (closed circle; *n* = 5), islet–FK506_M_ transplanted diabetic recipients on day 14 of transplantation (open triangle; *n* = 5), and islet–FK506_M_ transplanted diabetic recipients on day 28 of transplantation (closed triangle; *n* = 3). The values represent means ± SD.

We transplanted 2000 IEQ into the subcutaneous space over the flanks using Matrigel, with or without immune suppression. Non-fasting blood glucose (NBG) levels of the recipient mice were monitored after the transplantation. Diabetic mice without islet transplantation remained hyperglycemic and died before 10 days of induction (data not shown). Mice transplanted with islets remained euglycemic for less two weeks without immune suppression (median survival time, MST: 7.5 days) (Figure 3(b)). When the islets were codelivered with FK506_P_, the survival time of the transplanted islets was not increased (MST: 8 days) (Figure 3(c)). Based on our pharmacokinetics data, whole blood FK506 concentration in this group was higher than the recommended levels. Thus, we speculated an availability of high concentration of FK506 in the Matrigel might have impaired the viability and functionality of the transplanted islets. Encouragingly, the mice codelivered with islets and FK506_M_ remained euglycemic until the study period (survival rate: 60%), indicating a significant increase in the survival time of the transplanted islets compared to islet-only transplanted group (*p* = .00013) (Figure 3(d,f)). In contrast, transplantation of islets in one site and FK506_M_ on another site did not appreciably increase the survival time (Figure 3(e)). Furthermore, the blood glucose levels in the mice raised to hyperglycemic state within few days after the retrieval of the Matrigel containing the islets from the transplanted area. In addition, body weight of the mice dramatically decreased within few days after the retrieval of the Matrigel (Figure S3). This confirmed that the control in the blood glucose levels was solely due to the transplanted islets.

In addition, to evaluate the glucose responsiveness of the FK506_M_ group, IPGTT was performed on day 14 (*n* = 5) and day 28 (*n* = 3) of transplantation (Figure 3(g)). In both the normal and the FK506_M_ group, blood glucose levels gradually maintained to normoglycemic state within 60 min of intraperitoneal administration of 2 g/kg glucose solution. However, diabetic mice remained hyperglycemic over the sampling period. This indicated that the islet–FK506_M_ recipients had a normal response to an immediate rise in blood glucose.

### 3.7. Hematological analysis

Hematological tests were performed to evaluate the safety of FK506_M_ on the transplanted mice. Serum levels of blood urea nitrogen (BUN), creatinine (CRE), and electrolytes were measured to evaluate the renal function of the mice. As depicted in Table S1, BUN levels in islet–FK506_M_ recipients were significantly lower (25.3 ± 1.0 mg/dL) compared to the diabetic (33.5 ± 4.3 mg/dL) and other recipient groups (islet group: 30.3 ± 5.2 mg/dL and islet–FK506_P_ group: 29.7 ± 4.0 mg/dL). In addition, serum levels of Na^+^ in diabetic mice were significantly lower (*p* < .05 compared to normal mice). Transplantation of islet or islet–FK506_P_ did not satisfactorily improve the renal function of the recipients compared to the islet–FK506_M_ recipients. We did not observe further signs of renal toxicity associated with single administration of FK506_M_ at a dose of 10 mg/kg.

### 3.8. Quantification of insulin and TNF-α in serum and Matrigel

**Figure 4.**
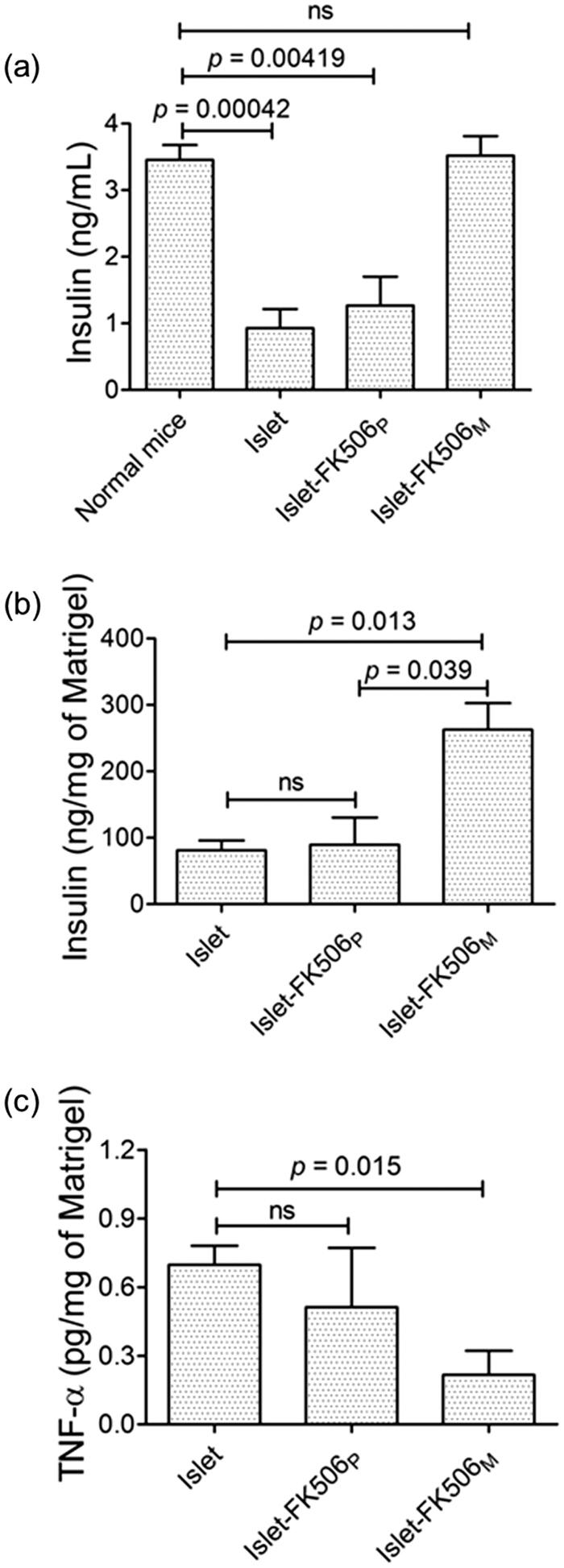
Measurement of insulin and proinflammatory cytokine levels in serum and Matrigel. (a) Serum insulin levels in different groups of mice. (b) Quantification of insulin per unit weight of Matrigel containing the transplanted islets after retrieval. (c) Quantification of TNF-α per unit weight of the Matrigel. In islet (*n* = 4) and islet–FK506_P_ (*n* = 5) recipients, the Matrigel was retrieved on day 15 post-transplantation. In islet–FK506_M_ recipients (*n* = 3), the Matrigel was retrieved on day 30 post-transplantation. TNF-α was not detected in the serum of any of the groups. The values represent means ± SD.

To evaluate the functionality of the transplanted islets, we quantified the levels of insulin in serum and Matrigel (Figure 4(a,b)). The level of insulin in islet–FK506_M_ (3.52 ± 0.51 ng/mL) was similar to that of normal mice (3.46 ± 0.44 ng/mL). However, significantly lower levels of insulin were detected in the serum of islet group (0.93 ± 0.57 ng/mL) (*p* = .00042) and islet–FK506_P_ group (1.21 ± 0.87 ng/mL) (*p* = .00419). Similarly, the insulin concentration in Matrigel of the islet–FK506_M_ group (262 ± 69.71 ng/mg of Matrigel) was significantly higher in comparison to the islet group (81.02 ± 25.45 ng/mg of Matrigel) (*p* = 0.013). These results confirmed the protection of the functionality of the islets codelivered with FK506_M_. Since the concentration of insulin in serum and Matrigel of islet–FK506_P_ recipients was significantly lower in comparison to that of islet–FK506_M_ (*p* = .039), we speculated a severe reduction in viability and functionality of islets due to high concentration of FK506 in the Matrigel.

In addition, we measured the levels of TNF-α to check the ability of FK506 for reducing the local inflammatory reactions in Matrigel and serum. As shown in Figure 4(c), the concentration of TNF-α was significantly lower (0.22 ± 0.18 pg/mg of Matrigel) in islet–FK506_M_ recipients compared to that of the islet-only recipients (0.70 ± 0.08 pg/mg of Matrigel) (*p* = .015). TNF-α was not detected in the serum, which suggested a negligible systemic inflammation after transplantation.

### 3.9. Immunohistochemistry (IHC)

**Figure 5.**
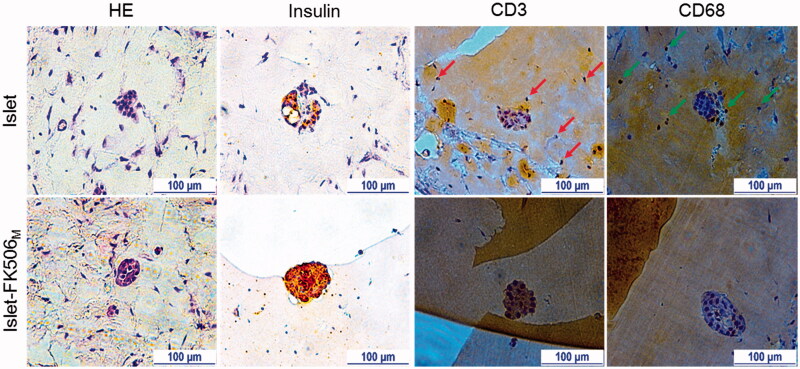
Immunohistochemistry analysis. H&E staining shows intact morphology of the islets inside the Matrigel of islet–FK506_M_ group. In addition, intense stain due to insulin was observed in the islet–FK506_M_ group compared to that of islet recipients. Higher proportion of CD3 (indicated by arrows) and CD68 (indicated by arrows) positive cells were observed in islet group compared to that of the islet–FK506_M_ group. In islet recipients, the Matrigel was retrieved on day 15 post-transplantation. In islet–FK506_M_ recipients, the Matrigel was retrieved on day 30 post-transplantation.

**Figure 6.**
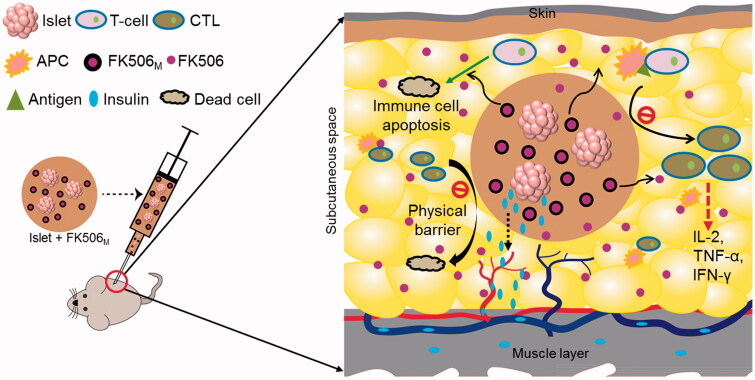
Schematic representation of immune protection protocol for local codelivery of pancreatic islets and FK506_M_. FK506 releases in a long-term from the microspheres inside the Matrigel containing islets. The release of FK506 at the local microenvironment inhibits the activation of T-cells. FK506 also has activity to cause apoptosis of T-cells. In addition, the solidified Matrigel may also act a physical barrier to massive infiltration of immune cells. The insulin secreted by the islets inside the hydrogel diffuses to the nearby blood vessels to reach the systemic circulation. CTL: cytotoxic T-lymphocytes; APC: antigen-presenting cell; FK506_M_: FK506-loaded poly(lactic-co-glycolic acid) microspheres.

To confirm the protective effects of FK506_M_ on the transplanted islets against host immune responses, Matrigels from different recipients were taken for histological analysis (Figure 5). At day 15 of transplantation, most of the islets in islet-only and islet-FK506_P_ recipients were damaged or had disappeared. In contrast, the islet morphology in islet–FK506_M_ recipients remained preserved for 30 days post-transplantation. IHC of insulin revealed the presence of sparse insulin stain in the gel of islet-only and islet–FK506_P_ recipients (Figure S5). In contrast, intense insulin stain was observed in the Matrigel of islet–FK506_M_ transplanted mice. Higher proportion of CD3 and CD68 positive cells was observed in the Matrigel of islet-only group (shown by red and green arrows, respectively) compared to that of islet–FK506_M_ transplanted mice. This indicated a strong inhibition of cytotoxic T-cell and macrophage proliferation in the Matrigel of islet–FK506_M_ recipients. Thus, codelivery of islets and FK506M successfully inhibited immune rejection (see Figure 6).

## 4. Discussion

Long-term survival of a graft necessitates the use of immune suppressive drugs. Activated T-cells play a crucial role in immune rejection in organ transplantation. These cells orchestrate the immune rejection cascade by releasing cytokines that regulate the proliferation and function of B-cells, macrophages, and other immune cells. Cytotoxic T-cells also attack and kill the graft cells directly (Crawley et al., 1997; Hamawy & Knechtle, 2003). To protect the transplanted islets from immune rejection, multiple studies report the potential application of locally-targeted immunosuppressive/immunomodulatory strategies. In this regard, authors have reported acceleration of islet engraftment when the dexamethasone-releasing macroporous scaffolds were used to promote anti-inflammatory M2 macrophages (Jiang et al., 2017). When pancreatic islets were codelivered with liposomal clodronate using an injectable hydrogel, the graft survival was significantly increased compared to that of islet-only transplanted group (Haque et al., 2014). Recently, we found an increase in the graft survival time when FK506-loaded nanoparticles were immobilized onto the surface of islet and transplanted into kidney capsule of immune-competent mice (unpublished data). In the present study, we fabricated microspheres that imparted prolonged-release profile of FK506 when injected into the subcutaneous space. FK506 is a macrolide antibiotic having potent immune suppressive properties to effectively block T-cell proliferation by inhibiting the production of IL-2, a growth factor needed for T-cell proliferation (Thomson et al., 1995). FK506 binds to FK506 binding protein (FKBP) and inhibits calcineurin phosphatase, leading to the inhibition of calcium-dependent pathways, such as IL-2 gene transcription, nitric oxide synthase activation, cell degranulation, and apoptosis (Thomson et al., 1995). It also leads to the impairment of helper T-cell mediated macrophage activation and cytotoxic T-cell mediated graft rejection (Thomson et al., 1995; Venkataramanan et al., 1995).

In this study, codelivery of pancreatic islets and FK506_M_ effectively suppressed immune rejection by inhibiting T-cell and macrophage proliferation in the local microenvironment of the graft. The subcutaneous space is avascular and has inadequate access of drugs from the systemic circulation, especially when the drugs bind to the blood cells. Due to availability of relatively high concentration of FK506 in the Matrigel, it can easily diffuse into the bloodstream due to sink condition of the body fluid. Once it reaches the systemic circulation, FK506 extensively binds to the red blood cells due to its excellent binding affinity (Piekoszewski et al., 1993; Karanam et al., 1998; Zahir et al., 2004). Due to the binding, systemically administered drug cannot reach the inadequately vascularized subcutaneous spaces. In our islet–FK506M^#^ group, a negligible concentration of FK506 might have diffused inside the Matrigel from the systemic circulation. As a result, graft survival time was not significantly increased. Based on previous findings, systemically administered FK506 has been reported to accumulate at higher concentration in liver, lungs, heart, and adrenal glands; a negligible amount of the drug was detected in skin and fat (Karanam et al., 1998). Systemic administration of FK506 has been used in the liver and kidney transplanted patients to produce effective immune suppression because of a high distribution of the drug in these organs (Li & Li, 2015).

On the other hand, systemic administration of FK506 for long-term immune suppression is associated with severe adverse drug reactions like hyperglycemia, hyperkalemia, and nephrotoxicity (Akar et al., 2005). Thus, strategies to provide local immune suppression may be considered attractive to prolong the survival of the grafts. In this regard, authors have reported 100% survival of allograft to more than 6 months with single dose local administration of FK506 disk in hind limb transplanted animals. Significant T-cell hyporesponsiveness was observed in local draining lymph nodes compared to that in the spleen of the single FK506 administered animals, indicating the effectiveness of FK506 in local immune suppression (Unadkat et al., 2017). Studies have also shown the prolonged allograft survival when the local draining lymph nodes were disrupted or removed (Barker & Billingham, 1968; Lakkis et al., 2000). Thus, targeting the draining lymph nodes may produce promising graft survival time without any systemic adversities. In another study, daily topical application of FK506 effectively prevented the skin graft by inhibiting the activation of immune cells in the skin (Solari et al., 2009). Topical application of FK506 effectively inhibited the infiltration of T-cells in the epidermis. It also inhibited expression of epidermal cytokines, impaired Th1 and Th2 cytokines, and suppressed the expression of costimulatory molecules (Homey et al., 1998). In our islet–FK506_M_ group, although we did not check the responsiveness of T-cells in the local draining lymph nodes, we speculated the accumulation of high concentration of FK506 in the draining lymph nodes might have either suppressed the activation of locally residing T-cells directly and/or inhibited the migration and maturation of antigen-presenting cells. Therefore, to achieve effective immune suppression in pancreatic islet transplantation, we suggest local immune suppression strategies such as, (1) development of a device for transplantation that can be periodically replenished with immune suppressive drug-encapsulated microspheres for prolonged-release of the drugs, (2) lymphadenectomy of the draining lymph node and a low dose immunosuppressive drug, and (3) low dose combination of immune suppressive drugs. The major challenge with clinical islet transplantation into the subcutaneous space is the lack of sufficient vascularization at the site. To protect the islets from hypoxic stress at the subcutaneous space, pre-vascularization at the site is necessary. We believe the codelivery of islets and low dose immune suppressant into the prevascularized subcutaneous space may overcome the need of frequent immune suppressive regimen in clinical islet transplantation. This will be the interest of our study in near future.

## 5. Conclusion

We established a protocol for local immune protection of transplanted islets with single subcutaneous injection of FK506_M_ using injectable hydrogel. In addition, we performed pharmacokinetics study and investigated renal function in the islet recipients to examine the safety of FK506_M_. The local delivery of FK506_M_ provided a sub-therapeutic level of the drug in the blood and effectively inhibited T-cell proliferation to block the immune rejection cascade mediated *via* macrophage activation. These findings have a great potential to develop a successful islet transplantation protocol in order to prevent acute graft rejection and to prolong the survival time of transplanted islets in type 1 diabetes.

## Supplementary Material

IDRD_Jeong_et_al_Supplemental_Comntent.docx
