# Supplementary material for: Single synchronous delivery of FK506-loaded polymeric microspheres with pancreatic islets for the successful treatment of streptozocin-induced diabetes in mice
Source: Drug Deliv. 2017 Sep 15;24(1):1350–9. doi: 10.1080/10717544.2017.1377317 (PMC8241191; doi:10.1080/10717544.2017.1377317)
Supplement: IDRD_Jeong_et_al_Supplemental_Comntent.docx [file IDRD_A_1377317_SM1374.docx]

**Supplementary information**

**Single synchronous delivery of FK506˗loaded polymeric microspheres with pancreatic islets for the treatment of streptozocin-induced diabetes in mice**

Shiva Pathak^1^, Shobha Regmi^1^, Biki Gupta^1^, Bijay K. Poudel^1^, Tung Thanh Pham^1^, Chul Soon Yong^1^, Jong Oh Kim^1^, Jae˗Ryong Kim^2^, Min Hui Park^3^, Young Kyung Bae^3^, Simmyung Yook^4^, Cheol˗Hee Ahn^5^, and Jee˗Heon Jeong^1,*^

*^1^College of Pharmacy, Yeungnam University, Gyeongsan, Gyeongbuk 38541, Republic of Korea*

*^2^Department of Biochemistry and Molecular Biology and Smart˗Aging Convergence Research Center, College of Medicine, Yeungnam University, Daegu 42415, Republic of Korea*

*^3^Department of Pathology, Yeungnam University College of Medicine, Daegu 42415, Republic of Korea*

*^4^College of Pharmacy, Keimyung University, Daegu 42601, Republic of Korea*

*^5^Engineering Research Institute, Department of Materials Science and Engineering, Seoul National University, Seoul 25354, Republic of Korea*

*Address for correspondence

Jee˗Heon Jeong

College of Pharmacy, Yeungnam University

280 Daehak˗ro, Gyeongsan˗si, Gyeongbuk˗do 38541, Republic of Korea

Tel: +82˗53˗810˗2822

E-mail: [jeeheon@yu.ac.kr](mailto:jeeheon@yu.ac.kr)

**Supplementary figures**


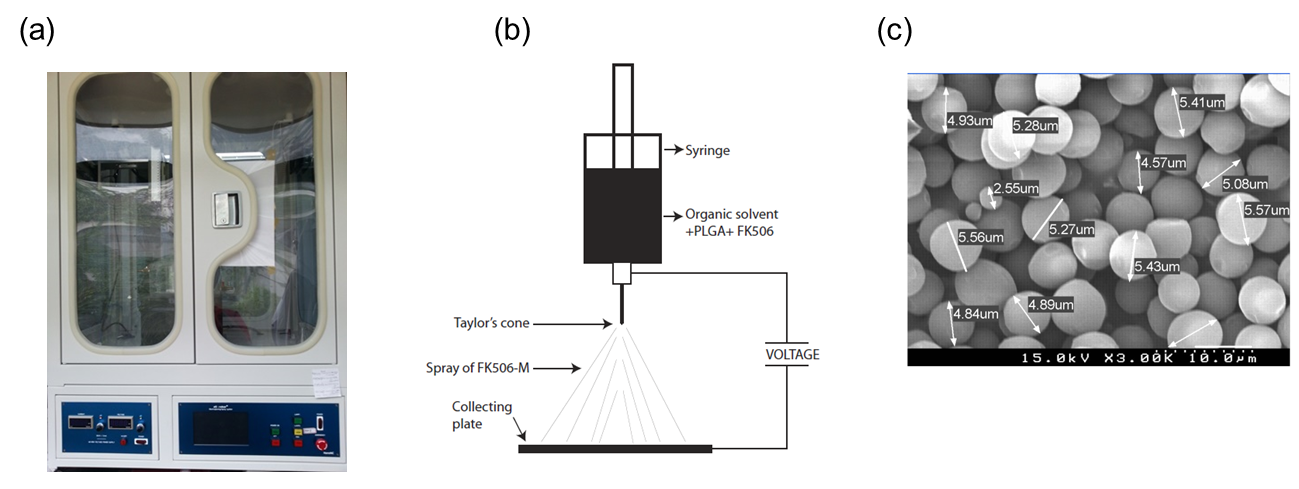


**Figure S1.** Preparation of FK506˗loaded microspheres (FK506_M_) using electrospray technique. (a) Optical image of electrospray machine. (b) Diagrammatic sketch of the principle of the electrospray machine. (c) Scanning electron microscopy (SEM) image of blank microspheres to show the diameters of microspheres using SEM software.


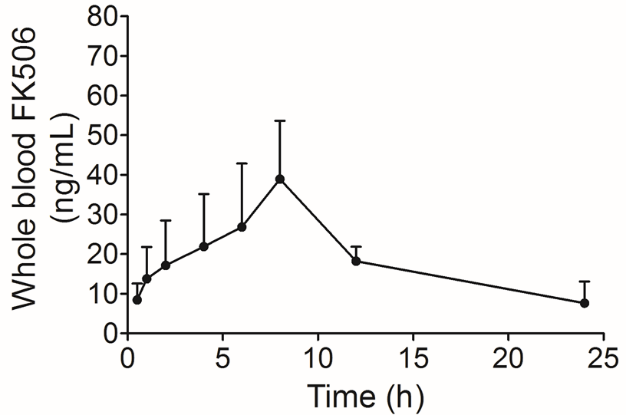


**Figure S2.** Pharmacokinetics of FK506 at a dose of 3 mg/kg in male Sprague Dawley rats. FK506 was dissolved in 40% PEG400 in PBS and was injected into the subcutaneous space.


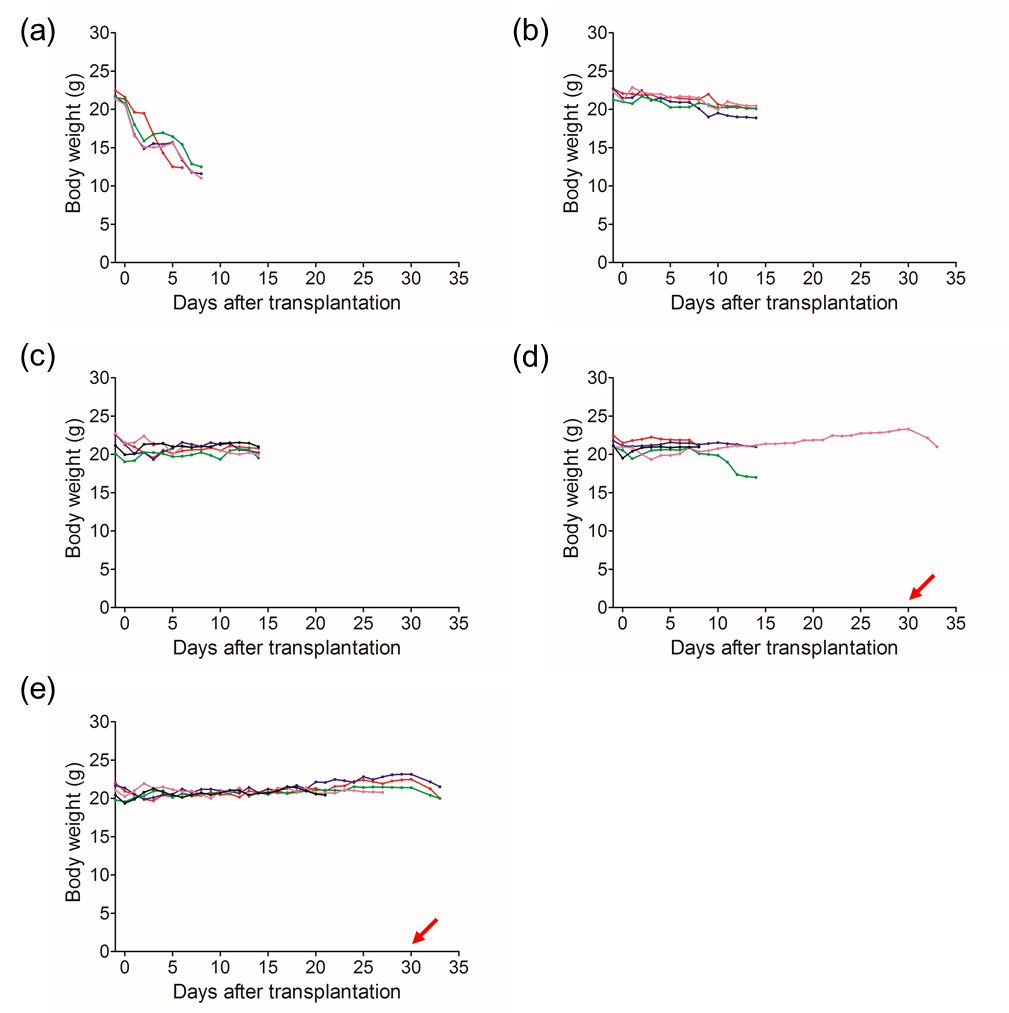


**Figure S3.** Body weight of different groups of mice. (a) Diabetic mice. (b) Islet˗only transplanted recipients. (c) Islet˗FK506_P_ transplanted recipients. (d) Islet˗FK506_M_ (different site) transplanted recipients. (e) Islet˗FK506_M_ (codelivery) transplanted recipients. Each line represents body weight of individual mouse. Red arrows at day 30 indicate the day of retrieval of islet containing Matrigel from the transplanted region. Day 0 indicates the day of islet transplantation. FK506_P_ indicates the powder form of FK506 and FK506_M_ indicated PLGA microspheres containing FK506.


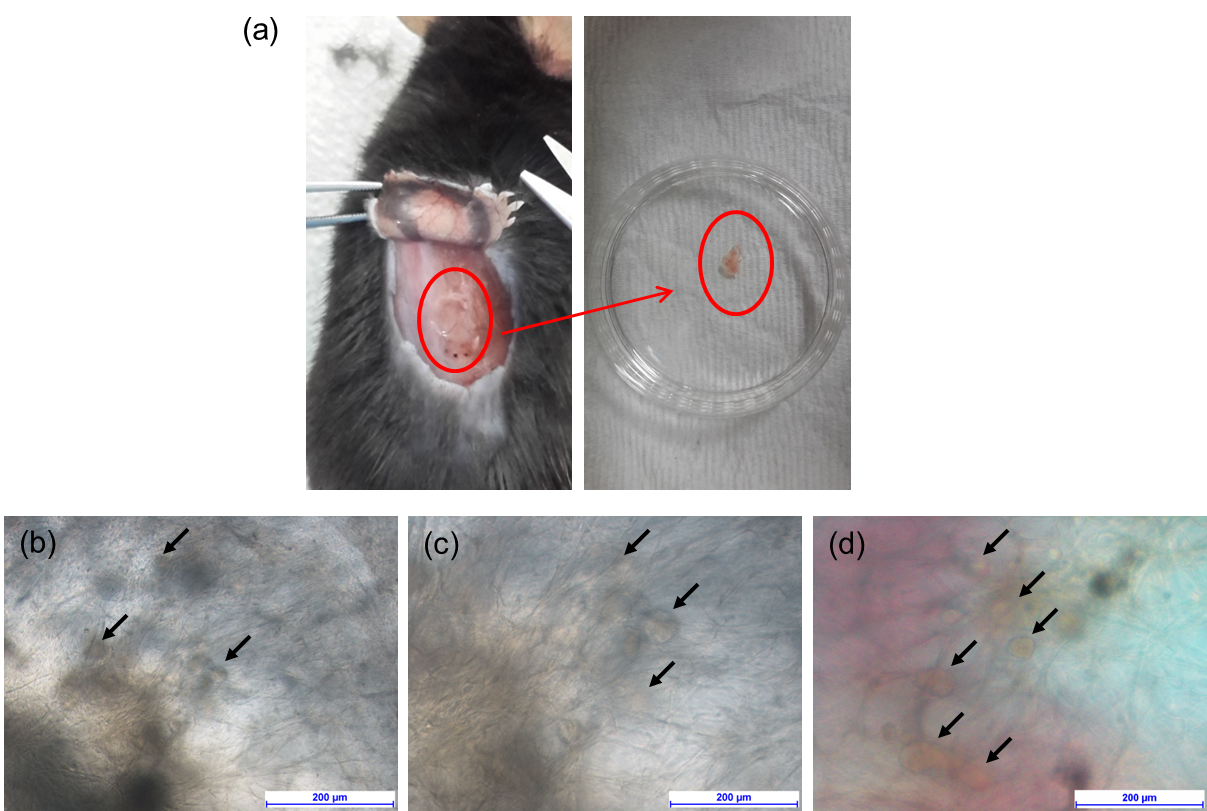


**Figure S4.** Retrieval of islet containing Matrigel from the transplanted recipients. Optical image of; (a) Matrigel localization in subcutaneous space of mice. (b) Matrigel of islet˗only recipient. (c) Matrigel of islet˗FK506_P_ recipient. (d) Matrigel of islet˗FK506_M_ recipient. Islets remained intact upto 30 days of post˗transplantation in islet˗FK506_M_ group. Numerous blood vessels were observed in islet˗FK506_M_ recipient (red color). Arrows indicate the islets inside the Matrigel.


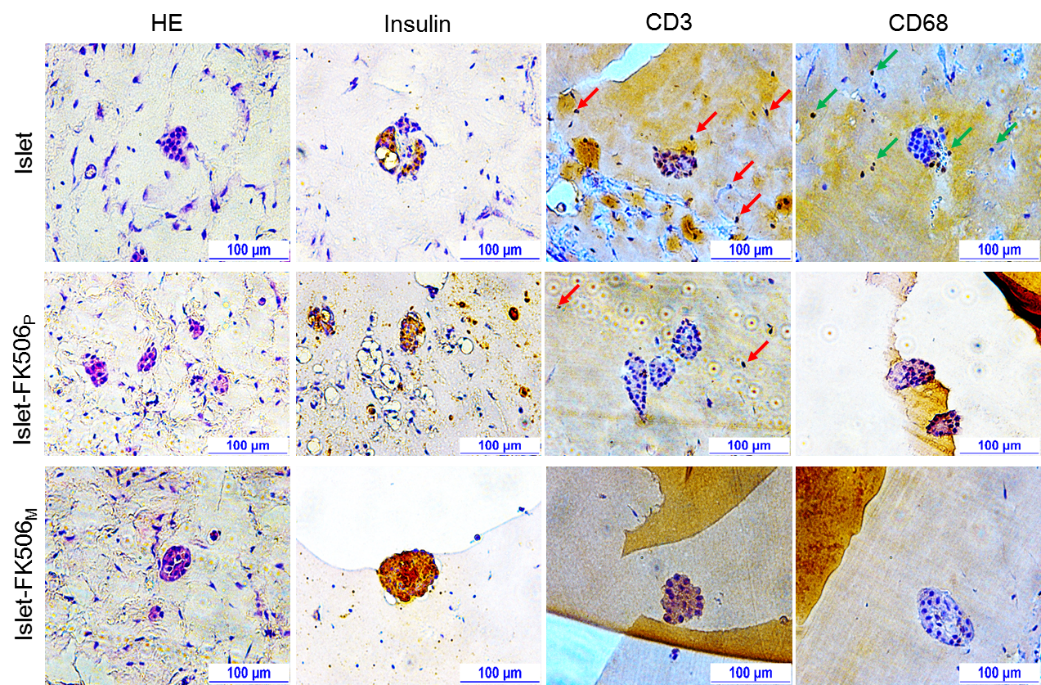


**Figure S5.** Immunohistochemistry analysis. HE staining shows intact morphology of islets inside Matrigel of islet˗FK506_M_ group. Intense stain due to insulin was observed in islet˗FK506_M_ group compared to that of control and islet-FK506_M_ groups. Higher proportion of CD3 (red arrows) and CD68 (green arrows) positive cells were observed in control compared to islet-FK506_P_ and islet˗FK506_M_ groups. In islet and islet-FK506_P_ recipients, the Matrigel was retrieved on day 15 post˗transplantation. In islet˗FK506_M_ recipients, the Matrigel was retrieved on day 30 post˗transplantation.

**Supplementary Table**

**Table S1.** Evaluation of renal function test in different group of mice

| Parameters | Normal mice | Diabetic mice | Islet˗only | Islet˗FK506_P_ | Islet˗FK506_M_ |
| --- | --- | --- | --- | --- | --- |
| BUN  CRE  Na^+^  K^+^  Cl^-^ | 20.1 ± 1.7  0.2 ± 0.1  148.3 ± 2.3  5.1 ± 1.3  112.3 ± 2.1 | 33.5 ± 4.3*  0.2 ± 0.1  137 ± 1.0*  6.9 ± 2.5  104.3 ± 4.5 | 30.3 ± 5.2*  0.2 ± 0.1  145.0 ± 1.3  5.1 ± 1.6  106.8 ± 4.9 | 29.7 ± 4.0*  0.2 ± 0.1  143.2 ± 1.1  4.3 ± 1.0  105.8 ± 0.8 | 25.3 ± 1.0  0.2 ± 0.0  145.0 ± 5.0  4.1 ± 0.6  105.3 ± 4.0 |

BUN: Blood urea nitrogen, CRE: Creatinine, Na^+^: Sodium, K^+^: Potassium, Cl^-^: Chloride

**Table S1.** Hematological analysis of different groups of mice. Blood sample was withdrawn to evaluate the kidney function test of mice. In islet˗only (n = 4) and islet-FK506_P_ (n = 5) transplanted recipients, blood sampling was performed on day 15 post˗transplantation. In islet˗FK506_M_ transplanted recipients (n = 3), the blood was withdrawn on day 30 post˗transplantation. The values represent means ± SD. *indicates *p* < 0.05 compared to normal mice (ANOVA).
